# Downregulation of Three Novel miRNAs in the Lymph Nodes of Sheep Immunized With the *Brucella suis* Strain 2 Vaccine

**DOI:** 10.3389/fvets.2022.813170

**Published:** 2022-02-22

**Authors:** Si Chen, Chengqiang Wang, Qiaoling Chen, Dantong Zhao, Yongbin Liu, Shihua Zhao, Shaoyin Fu, Xiaolong He, Bin Yang, Qinan Zhao, Qi An, Zhenxing Zhang, Yiwen Cheng, Churiga Man, Guoying Liu, Xuefeng Wei, Wenguang Zhang, Li Du, Fengyang Wang

**Affiliations:** ^1^Hainan Key Lab of Tropical Animal Reproduction, Animal Genetic Engineering Key Lab of Haikou, Breeding and Epidemic Disease Research, College of Animal Science and Technology, Hainan University, Haikou, China; ^2^Jinyu Baoling Bio-Pharmaceutical Co., Ltd., Hohhot, China; ^3^Inner Mongolia University, Hohhot, China; ^4^Inner Mongolia Academy of Agricultural and Animal Husbandry Sciences, Hohhot, China; ^5^College of Animal Science, Inner Mongolia Agricultural University, Hohhot, China

**Keywords:** *Brucella*, sheep, lymphatic nodes, miRNAs, downregulation

## Abstract

Ovine and caprine brucellosis, both caused by *Brucella melitensis*, lead to substantial economic losses in the animal industry and health problems in human populations. *Brucella suis* strain 2 (*B.suis S2*), as a live attenuated vaccine, is used extensively in China to prevent brucellosis. It has been proven that microRNA (miRNAs) are involved in the immunopathogenesis of brucellosis; however, the miRNA-driven mechanism of immune response to *B.suis* S2 *in vivo* remains unknown. To determine which new miRNAs are involved in the host immune response to *B.suis* S2 and elucidate the function of these miRNAs, we performed a comprehensive analysis of miRNA expression profiles in sheep immunized with *B.suis* S2 using the high-throughput sequencing approach. The submandibular lymphatic nodes from sheep seropositive for *Brucella* were collected at 7, 14, 21, 30, 60 and 90 days post-immunization. MiRNA sequencing analysis revealed that 282 differentially expressed miRNAs (|log_2_ fold-change |>0.5 and *p* < 0.05) were significantly enriched in the immune pathways, including the NF-kappa B signaling pathway, B cell receptor signaling pathway, p53 signaling pathway and complement and coagulation cascades. Increasing the threshold to |log_2_ fold change|>1 and *p* < 0.01 revealed 48 differentially expressed miRNAs, 31 of which were novel miRNAs. Thirteen of these novel miRNAs, which were differentially expressed for at least two time points, were detected via RT-qPCR assays. The novel_229, novel_609, novel_973 and oar-miR-181a assessed by RT-qPCR were detectable and consistent with the expression patterns obtained by miRNA sequencing. Functional analyses of these miRNAs demonstrated that their target genes participated in the immune response pathways, including the innate and adaptive immunity pathways. The immune-related target genes of novel_229 included *ENSOARG00000000649* and *TMED1*, as well as *LCN2, PDPK1* and *LPO* were novel_609 target genes. The immune-related target genes of novel_973 included *C6orf58, SPPL3, BPIFB1, ENSOARG00000021083, MPTX1, CCL28, FGB, IDO1, OLR1* and *ENSOARG00000020393*. The immune-related target genes of oar-miR-181a included *ENSOARG00000002722, ARHGEF2, MFAP4 and DOK2*. These results will deepen our understanding of the host miRNA-driven defense mechanism in sheep immunized with *B.suis* S2 vaccine, and provide the valuable information for optimizing vaccines and developing molecular diagnostic targets.

## Introduction

Brucellosis, caused by infection with *Brucella* spp., is among the most common bacterial zoonoses. *Brucella* spp. infect both humans and non-human animals, including sheep, goats, cattle, swine and a variety of feral animals. Globally, over half a million humans are infected annually (1). Malta fever, chronic debilitating disease, spontaneous abortions and sterility are the main clinical symptoms of brucellosis. The disease tends to be chronic and persistent, which causes its host to become febrile and masquerades as many other diseases (2). Brucellosis in domestic animals, especially in ovine and caprine herds, causes adverse economic effects on animal production and affects public health (3). Vaccine inoculation is an effective preventive measure. Since 1971, the *Brucella suis* strain 2 (*B.suis* S2) vaccine has been used successfully in China to orally vaccinate sheep and goats against brucellosis (4).

Recently, studies have increasingly reported the mechanisms of *B.suis* S2 vaccine-elicited immune protection. On the one hand, focusing on *Brucella*, the comparative genomic analyses of *B.suis* S2 were conducted to study their genetic basis for virulence attenuation. Di and his colleagues found that *eryD* and *OmaA* genes in the *B.suis* S2 genome were related to the virulence attenuation (5). On the other hand, focusing on the host, the immune responses of different host cells infected with *B.suis* S2 were analyzed, which has prompted the further application of this vaccine in a natural host. Transcriptome analysis of murine macrophages infected with *B.suis* S2 revealed that 11 differently expressed genes were related to the cellular responses to lipopolysaccharide pathway and were significantly upregulated in *B.suis* S2-infected macrophages (6). Wang et al. observed that the replication of *B.suis* S2 induced goat trophoblast cell apoptosis and growth retardation under endoplasmic reticular stress (7). In human microglia clone 3 (HMC3) cells, *B.suis* S2 inhibited the JNK/p53 signaling pathway and suppressed apoptosis by increasing CALR protein expression (8). Recent transcriptomic analysis of peripheral blood mononuclear cells from monkeys immunized with the S2 vaccine showed that most downregulated genes were related to cell adhesion molecules, cytokine-cytokine receptor interaction, and chemokine signaling pathways (9). However, knowledge of the molecular mechanisms involved in vaccine-mediated protection *in vivo* remains incomplete, especially in ovine herds.

MicroRNAs (miRNAs) are endogenous, noncoding RNAs that regulate the post-transcriptional expression of mRNAs. It has been stated that miRNAs have a crucial role in the immune evasion of *Brucella* spp (10). Recent studies have shown that miRNAs, such as miR-125b-5p and miR-24, can activate the NF-kappa B (NF-κB) pathway and inhibit the expression of the *STING* gene, thereby influencing bacterial intracellular survival (11, 12). In *Brucella*-infected macrophages, the downregulation of miR-21-5p reduced *IL-10* gene expression and increased the expressions of GBP5 and IL-12, thus inhibiting *Brucella* intracellular growth (13, 14). The miR-181 family has been proven to be dynamically regulated during T-cell development and activation (15). During the *B. suis* 1330 infection, miR-181a-5p expression was induced in porcine and murine macrophages, suggesting that miR-181a-5p may be involved in adaptive immunity (16). Additionally, Zhang et al. identified high miR-103b expression levels in the sera of patients with confirmed brucellosis, providing a reference for the auxiliary diagnoses of brucellosis infections (17).

We previously found that infecting RAW264.7 macrophages with *B. melitensis* Δ*Omp25* altered the expressions of miR-149-3p and miR-146a-5p, and the target genes of miR-149-3p were related to the host immune responses, autophagy and phagosome maturation (18). After infection with *B. melitensis* M5-90 Δper, the increased expression of miR-146b-5p inhibited autophagy activation in RAW264.7 cells (19). In goat fibroblasts, we presented a comprehensive miRNA profile in response to *B. melitensis* M5-90 infection and observed that three miRNAs (miR-744, miR-29a-5p and miR-193b-5p) played pleiotropic roles in inflammatory and immune responses (20). Most organisms contain numerous miRNAs; for example, ~1000 miRNAs were identified within the human genome (21). However, our understanding of ovine miRNAs is very limited. Using next-generation sequencing technology, Wong et al. detected 353 miRNAs in high abundance. Among them, 89 were the novel ovine miRNAs, which showed 70–99% alignment with hsa-, mmu- and/or rno-miRNAs, and 78 were the previously known oar-miRNAs (in miRBase v21) (22). The lack of information limits determining the immune mechanisms of sheep against pathogens and vaccines, including *B.suis* S2.

Given the knowledge of miRNAs associated with immunological processes, we hypothesized that miRNAs might also play key roles in *B.suis* S2 vaccine-induced immunity *in vivo*. Here, we performed clinical trials using sheep vaccinated with the *B.suis* S2 vaccine and monitored them for 90 consecutive days post-immunization (dpi). The high-throughput miRNA sequencing (miRNA-seq) technology were applied to reveal the dynamic miRNA expression profiles from the lymph nodes of vaccinated sheep focusing on the novel miRNAs. We identified three novel downregulated miRNAs involved in the immune response. We aimed to identify the roles of these novel ovine miRNAs in the immune responses to the *B.suis* S2 vaccination and provide valuable information for vaccine research on brucellosis.

## Materials and Methods

### Animals and Ethics Statement

**Figure 1:**
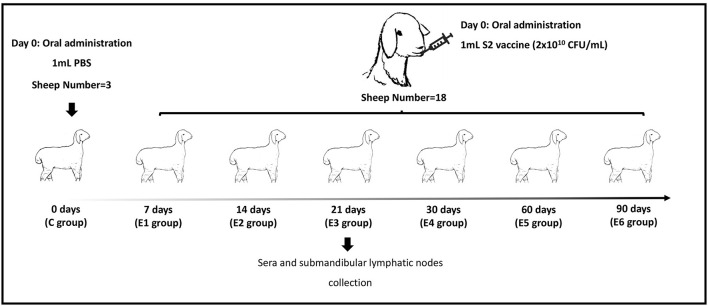
Experiment strategy. C represents the control group. E1–E6 represent 7, 14, 21, 30, 60 and 90 days post-immunization groups.

Twenty-one 10-month-old unmated female sheep serologically free of *Brucella*-specific antibodies were transported to the Biosafety Level 3 (BSL-3) laboratory of Jinyu Baoling Bio-pharmaceutical Co., Ltd (Inner Mongolia, China) for experiment. Sheep were serologically tested using the Pourquier^®^ Rose Bengal Brucellosis Antigen (IDEXX, P00215, ME, USA) and AsurDx^TM^ Brucella Multispecies Antibodies cELISA Test Kit (BIOSTONE, 10043-05, TX, USA). Sheep were kept in raised cages separately to prevent cross-transmission of infection and were offered *ad libitum* feeding. Animals were randomly divided into seven groups (*n* = 3): one control group (C group) and six groups inoculated with *Brucella. suis* strain S2 (E1 group to E6 group). At indicated time points post infection, sheep were slaughtered humanely (Figure 1). All animal procedures were conducted in accordance with the Regulations for the Administration of Affairs Concerning Experimental Animals (Ministry of Science and Technology, China, 2004) and were approved by the Academic Committee of the College of Animal Science and Technology of Hainan University as per the regulations on the use of experimental animals and institutional safety procedures.

### Oral Immunization and Sample Collection

In the control group, three sheep were inoculated with 1 mL sterilized phosphate-buffered saline (PBS, pH 7.2). The remaining 18 sheep were immunized with the lyophilized *B.suis* S2 vaccine (with an amount of 2 × 10^10^ CFU) via oral administration. The number of colony-forming units (CFUs) of *B.suis* S2 was determined by plating serial dilutions on plate count agar plates and 2 × 10^10^ CFU in 1 mL sterilized PBS (pH 7.2) was used. The vaccine was provided by Jinyu Baoling Bio-pharmaceutical Co., Ltd (Inner Mongolia, China). As shown in Figure 1, the ovine submandibular lymphatic nodes from the control group and vaccinated groups were collected at 0, 7, 14, 21, 30, 60 and 90 days post-immunization (dpi) individually. RNA was extracted using the standard TRIzol (15596026, Invitrogen, USA) reagent protocol, and DNase I was used to remove contaminating gDNA. RNA degradation and contamination were monitored on 1% agarose gels and checked using the NanoPhotometer^®^ spectrophotometer (IMPLEN, CA, USA). The RNA concentration was measured using the Qubit^®^ RNA Assay Kit in Qubit^®^ 2.0 Fluorometer (Life Technologies, CA, USA). RNA integrity was assessed using the RNA Nano 6000 Assay Kit of the Agilent Bioanalyzer 2100 system (Agilent Technologies, CA, USA). The raw reads were deposited to the NCBI Sequence Read Archive under accession no. PRJNA775839.

### Quantification of *Brucella* Antibodies in Serum

Ovine serum samples were collected prior to the first immunization and at 7, 14, 21, 30, 60, and 90 dpi. Serum samples were tested using the Pourquier^®^ Rose Bengal Brucellosis Antigen (IDEXX, P00215, ME, USA) and AsurDx^TM^ Brucella Multispecies Antibodies cELISA Test Kit (BIOSTONE, 10043-05, TX, USA). In the Rose Bengal Test (RBT) assay, 25 μL of each serum was dispensed on the plates. The same volume of Rose Bengal Brucellosis Antigen was added beside each sample. The serum and Rose Bengal Brucellosis Antigen were mixed to produce a circular 2 cm in diameter. The plates were shaken gently for 4 min to read the results. The competitive ELISA (cELISA) tests were carried out on 96-well microplates. In each well, 10 μL of serum was added to 40 μL of 1 × diluent solution to obtain a 1:5 dilution. Then, 50 μL of 1 × Anti-Brucella Antibody-HRP Conjugate was added. Finally, after 1 h incubation and washing, the reaction was revealed by adding 100 μL/well of TMB substrate and incubated at room temperature in the dark for 15 min. 100 μL of Stop Solution was added to each well of the plate. The OD value was read at 450 nm wavelength in a Multiskan^®^ FC reader (Thermo Fisher Scientific Inc,). Each serum was processed in quadruplicate. Results were analyzed in terms of Percentage of inhibition (PI), defined as: PI = (1-OD_450_ test sample / mean OD_450_ negative control)^*^100.

### Small RNA Sequencing and Normalization

3 μg total RNAs per sample were used to construct the small RNA libraries. Sequencing libraries were generated using NEBNext^®^ Multiplex Small RNA Library Prep Set for Illumina^®^ (New England Biolabs, Beverly, MA, USA), and index codes were added to attribute sequences to each sample. The library quality was assessed on the Agilent Bioanalyzer 2100 system. Index-coded samples were clustered on a cBot Cluster Generation System using TruSeq SR Cluster Kit v3-cBot-HS (GD-401-3001, Illumina, CA, USA). After cluster generation, the library preparations were sequenced on an Illumina HiSeq 2500/2000 platform to generate 50-bp single-end reads. Raw data in fastq format was first processed through custom perl and python scripts. In this step, clean reads were obtained by removing reads containing poly-N, with 5′ adapter contaminants, without the 3′ adapter or the insert tag, containing poly A or T or G or C and low-quality reads from the raw data. Q20, Q30, and the GC content of the raw data were calculated. The genome assembly corresponded to GenBank Assembly ID GCA_000298735.1. A specified portion of the clean reads was used to perform all downstream analyses. The small RNA tags were mapped to the reference sequences in Bowtie (23) without mismatches to analyze their expressions and distributions on the reference. To remove tags originating from protein-coding genes, repeat sequences, rRNA, tRNA, snRNA, and snoRNA, small RNA tags were mapped to RepeatMasker, the Rfam database or data from the specified species. Mapped small RNA tags were used to look for known miRNA. miRBase20.0 was used as a reference, and the modified software miRDeep2 (24) and srna-tools-cli were used to obtain the potential miRNA and draw the secondary structures. The unannotated sRNAs, which had the characteristics of the hairpin structure of the miRNA precursor, were used to predict novel miRNAs via miREvo (25) and miRDeep2. Custom scripts were used to obtain the identified miRNA counts and base biases on the first position with a certain length and on each position of all identified miRNAs. The miRNA read counts were normalized as transcripts per million based on the formula: normalized expression = mapped read count/total reads ×1,000,000 (26).

### Differential Expression and Enrichment Analysis

Differential expressions of two groups were analyzed using the DESeq R package (1.8.3). *P*-values were adjusted using the Benjamini-Hochberg method. A default corrected *P*-value of 0.05 was set as the threshold for significantly differential expressions. In order to identify the function of these miRNAs, the candidate target genes of differentially expressed miRNAs were analyzed by Gene Ontology (GO) and Kyoto Encyclopedia of Genes and Genomes (KEGG) systems. The Wallenius' non-central hyper-geometric distribution, which adjusts the gene length bias, was implemented for GO enrichment analysis (27). KEGG is a database resource for understanding high-level functions and utilities of biological systems, such as cells, organisms and ecosystems, from molecular-level information, especially large-scale molecular datasets generated by genome sequencing and other high-throughput experimental technologies (http://www.genome.jp/kegg/) (28). KOBAS software was used to test the statistical enrichment of the target genes in KEGG pathways (29).

### Novel miRNAs and Target Gene Identification

RT-qPCR was used to verify the reliability of miRNA sequencing. Total RNAs were isolated from the submandibular lymphatic nodes by using TRIzol reagent (15596026, Invitrogen, USA) following the manufacturer's protocol. The left and right submandibular lymphatic nodes of sheep were collected, respectively. In the E4 group, the right submandibular lymphatic node of No.244 sheep was deficient. The miRcute Plus miRNA First-Strand cDNA Kit (KR211, TIANGEN, Beijing, China) was used to reverse transcribe total RNAs. RT-qPCR was conducted following the protocol from the miRcute Plus miRNA qPCR Kit (FP411, TIANGEN, Beijing, China). The specific stem-loop reverse primer sequence was 5′-AGTGCAGGGTCCGAGGTA-3′. The expression levels of target miRNAs were normalized to *U6*. Relative transcriptional levels were determined using the 2^−Δ*ΔCt*^ method. The experiments were repeated three times. All RT-qPCR results are expressed as the mean ± standard error of the mean. *T*-tests were performed on the RT-qPCR data to determine whether the difference was statistically significant (30). *P* < 0.05 was considered statistically significant. Target genes of the differentially expressed miRNAs were predicted via Targetscan (-cps 50 -en−10), miRanda (-sc 140 -en−10 -scale 4 -strict) and RNAhybrid (-e−10 -p 0.05 -m 50000). After applying these filters, the residual miRNAs and target genes were used to construct miRNA-mRNA networks using Cytoscape 3.8.2 software.

## Results

### Detection of *B. suis* Strain 2 Antibodies in Serum

The ovine sera were collected for serological testing 1 day before inoculation and at 7, 14, 21, 30, 60 and 90 dpi. Table 1 compares the results of RBT and cELISA obtained with the serum of 21 sheep. The specific anti-*B. suis* IgG antibody was detected in the sera of sheep immunized with *B.suis* S2, whereas no specific reactivity was observed in the control group. *B. suis* antigen-specific serum IgG was detected by RBT and cELISA at the 7 dpi. The IgG antibodies peaked by day 14 after the first immunization and remained the level from 14 dpi to 90 dpi (Table 1). These results demonstrated that *B.suis* S2 was able to elicit the *B. suis* specific antibody response.

**Table 1:** Results of Rose Bengal Test (RBT) and cELISA in sheep (*n* = 21) with *B.suis* S2.

**Sheep** **name**	**Days post-immunization** **(dpi)**	**RBT** **result**	**cELISA** **PI value** **(mean ±SD)**
C_379		-	23.73 ± 6.12
C_515	0	-	36.37 ± 3.27
C_587		-	36.30 ± 7.57
E1_239		-	22.79 ± 1.64
E1_273	7	-	64.09 ± 4.59
E1_283		+	81.71 ± 2.56
E2_231		++	87.63 ± 1.42
E2_237	14	++	87.25 ± 2.07
E2_265		+++	88.76 ± 1.83
E3_221		+++	86.49 ± 1.08
E3_285	21	+++	87.84 ± 1.23
E3_292		++	87.37 ± 1.45
E4_204		++	85.34 ± 1.97
E4_215	30	+++	89.11 ± 1.06
E4_244		+++	83.49 ± 2.15
E5_254		+++	83.27 ± 2.21
E5_263	60	+++	89.32 ± 2.29
E5_280		+++	90.55 ± 1.25
E6_214		++	88.50 ± 0.24
E6_268	90	+	81.37 ± 2.36
E6_270		+++	88.79 ± 1.43

*“-” revealed that the tested sera by RBT was negative for Brucella antibodies, while “+” revealed the positive reactions for Brucella antibodies. “+”, “++” and “+++” revealed the different degrees of agglutination in RBT, from low to high*.

### Overview of Total Small RNA Libraries

**Figure 2:**
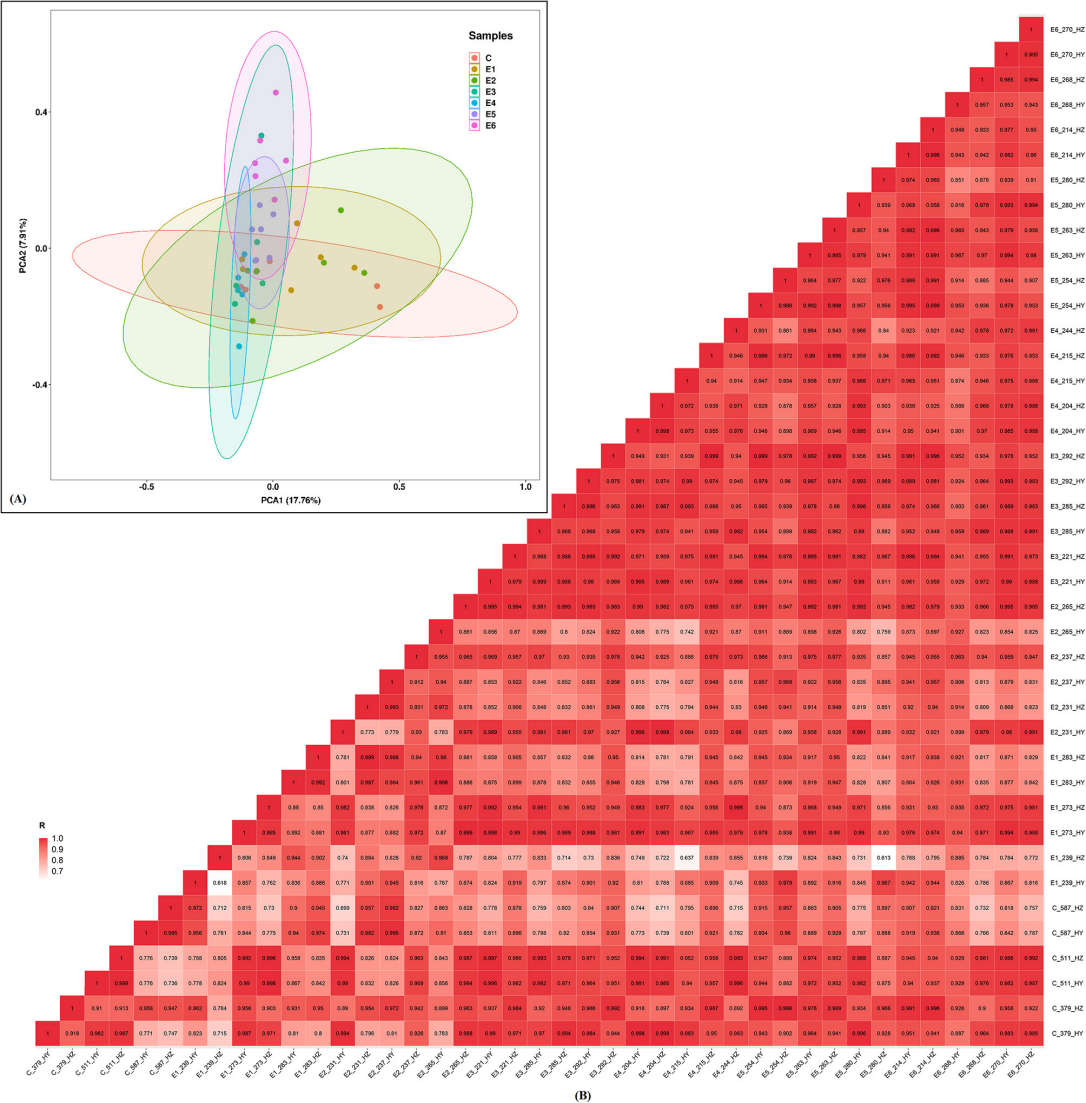
Principal component analysis (PCA) plot and correlation heatmap. **(A)** PCA score plot. Different colors denote the investigated time points. **(B)** Correlation heatmap of samples. The gradient color barcode at the right indicates the minimum value in white and the maximum in red. If one sample is highly similar to another one, the correlation value between them is very close to 1.

The RNA integrity number (RIN) of each RNA sample was above 6.0, which indicated these RNA can be used for further constructing small RNA libraries. The sequencing quality score (Q30), which represent the base call accuracy of 99.90%, were above 93.49% for each sample, signifying that the sequencing quality was sufficient for subsequent sequence assembly (Supplementary Table 1). The mapped ratios of the clean reads were more than 86.65% and the numbers of mapped mature miRNAs were between 105 and 132 (Table 2). Small RNAs (sRNAs) ranging from 18 to 30 nt long were further analyzed and the unannotated sRNAs were selected to predict novel miRNAs via miREvo and miRDeep2. Principal components analysis showed that the first principal component accounted for 17.76% of the variance, and the second principal component accounted for 7.91% of the variance. The clustering data points were clearly visible among the groups (Figure 2A). The correlation heatmap revealed good agreement among the groups (Figure 2B).

**Table 2:** The sRNAs mapped results.

**Sample**	**Total reads**	**Clean reads**	**Mapped total sRNAs**	**Mapped mature miRNAs**
C_379_HY	15,201,907 (100.00%)	13,172,378 (86.65%)	7,299,277 (48.02%)	117
C_379_HZ	16,857,634 (100.00%)	15,781,374 (93.62%)	7,848,504 (46.56%)	123
C_511_HY	14,494,014 (100.00%)	14,034,350 (96.83%)	7,519,694 (51.88%)	121
C_511_HZ	16,239,452 (100.00%)	15,914,002 (98.00%)	8,395,143 (51.7%)	117
C_587_HY	14,045,769 (100.00%)	13,240,698 (94.27%)	5,075,481 (36.14%)	127
C_587_HZ	22,478,306 (100.00%)	22,180,312 (98.67%)	8,841,773 (39.33%)	132
E1_239_HY	15,498,163 (100.00%)	14,848,429 (95.81%)	6,188,333 (39.93%)	110
E1_239_HZ	10,994,015 (100.00%)	10,215,799 (92.92%)	4,052,406 (36.86%)	124
E1_273_HY	16,486,598 (100.00%)	15,915,768 (96.54%)	10,355,334 (62.81%)	121
E1_273_HZ	16,714,776 (100.00%)	16,373,491 (97.96%)	9,584,909 (57.34%)	120
E1_283_HY	16,949,035 (100.00%)	15,921,999 (93.94%)	7,078,423 (41.76%)	130
E1_283_HZ	15,672,091 (100.00%)	15,342,060 (97.89%)	5,201,947 (33.19%)	128
E2_231_HY	14,242,129 (100.00%)	13,857,044 (97.30%)	8,033,474 (56.41%)	125
E2_231_HZ	14,801,355 (100.00%)	13,921,249 (94.05%)	5,800,471 (39.19%)	132
E2_237_HY	15,694,994 (100.00%)	15,459,256 (98.50%)	6,810,509 (43.39%)	129
E2_237_HZ	16,226,236 (100.00%)	14,281,640 (88.02%)	7,350,952 (45.3%)	116
E2_265_HY	14,110,328 (100.00%)	13,911,529 (98.59%)	5,730,447 (40.61%)	129
E2_265_HZ	16,128,226 (100.00%)	15,660,136 (97.10%)	9,470,821 (58.72%)	127
E3_221_HY	15,353,429 (100.00%)	15,057,972 (98.08%)	9,851,865 (64.17%)	113
E3_221_HZ	16,726,967 (100.00%)	16,119,367 (96.37%)	8,759,290 (52.37%)	112
E3_285_HY	15,437,776 (100.00%)	15,081,709 (97.69%)	7,469,083 (48.38%)	122
E3_285_HZ	14,808,719 (100.00%)	14,242,649 (96.18%)	9,766,214 (65.95%)	116
E3_292_HY	16,509,571 (100.00%)	16,199,891 (98.12%)	8,406,951 (50.92%)	120
E3_292_HZ	16,891,657 (100.00%)	16,142,634 (95.57%)	8,737,141 (51.72%)	118
E4_204_HY	15,185,208 (100.00%)	14,416,274 (94.94%)	9,280,198 (61.11%)	123
E4_204_HZ	16,276,393 (100.00%)	15,522,899 (95.37%)	10,089,616 (61.99%)	125
E4_215_HY	14,613,512 (100.00%)	14,069,221 (96.28%)	8,616,207 (58.96%)	118
E4_215_HZ	16,261,830 (100.00%)	14,645,087 (90.06%)	9,215,475 (56.67%)	105
E4_244_HZ	16,610,508 (100.00%)	16,371,786 (98.56%)	9,034,705 (54.39%)	121
E5_254_HY	15,312,223 (100.00%)	14,682,307 (95.89%)	7,510,388 (49.05%)	122
E5_254_HZ	14,460,756 (100.00%)	14,111,285 (97.58%)	6,520,178 (45.09%)	117
E5_263_HY	16,665,351 (100.00%)	16,444,759 (98.68%)	8,017,408 (48.11%)	117
E5_263_HZ	15,533,490 (100.00%)	15,328,090 (98.68%)	7,436,524 (47.87%)	123
E5_280_HY	15,653,751 (100.00%)	15,424,987 (98.54%)	7,816,125 (49.93%)	121
E5_280_HZ	15,706,737 (100.00%)	15,335,614 (97.64%)	7,446,763(47.41%)	119
E6_214_HY	14,366,059 (100.00%)	13,876,706 (96.59%)	6,830,523 (47.55%)	129
E6_214_HZ	15,163,527 (100.00%)	15,001,253 (98.93%)	7,516,219 (49.57%)	123
E6_268_HY	14,755,250 (100.00%)	14,625,057 (99.12%)	6,965,737 (47.21%)	118
E6_268_HZ	15,468,239 (100.00%)	15,332,475 (99.12%)	7,467,237 (48.27%)	123
E6_270_HY	14,202,828 (100.00%)	14,027,069 (98.76%)	7,310,328 (51.47%)	122
E6_270_HZ	14,292,165 (100.00%)	14,148,409 (98.99%)	7,413,842 (51.87%)	122

### Novel miRNAs Screening and Differentially Expressed miRNAs Identification

**Figure 3:**
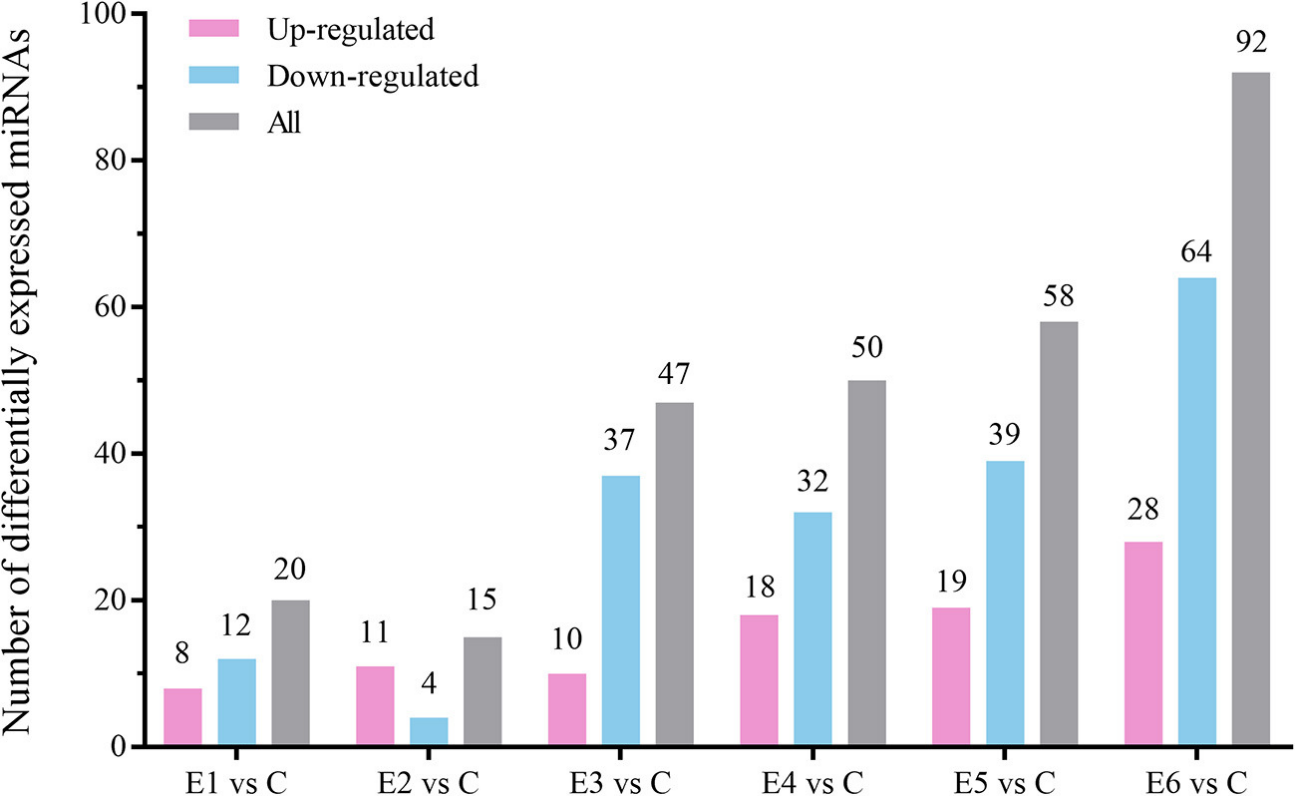
The number of up-regulated and down-regulated differentially expressed miRNAs at different time points compared with the control group (|log_2_ (fold-change)| > 0.5, *p* < 0.05).

**Figure 4:**
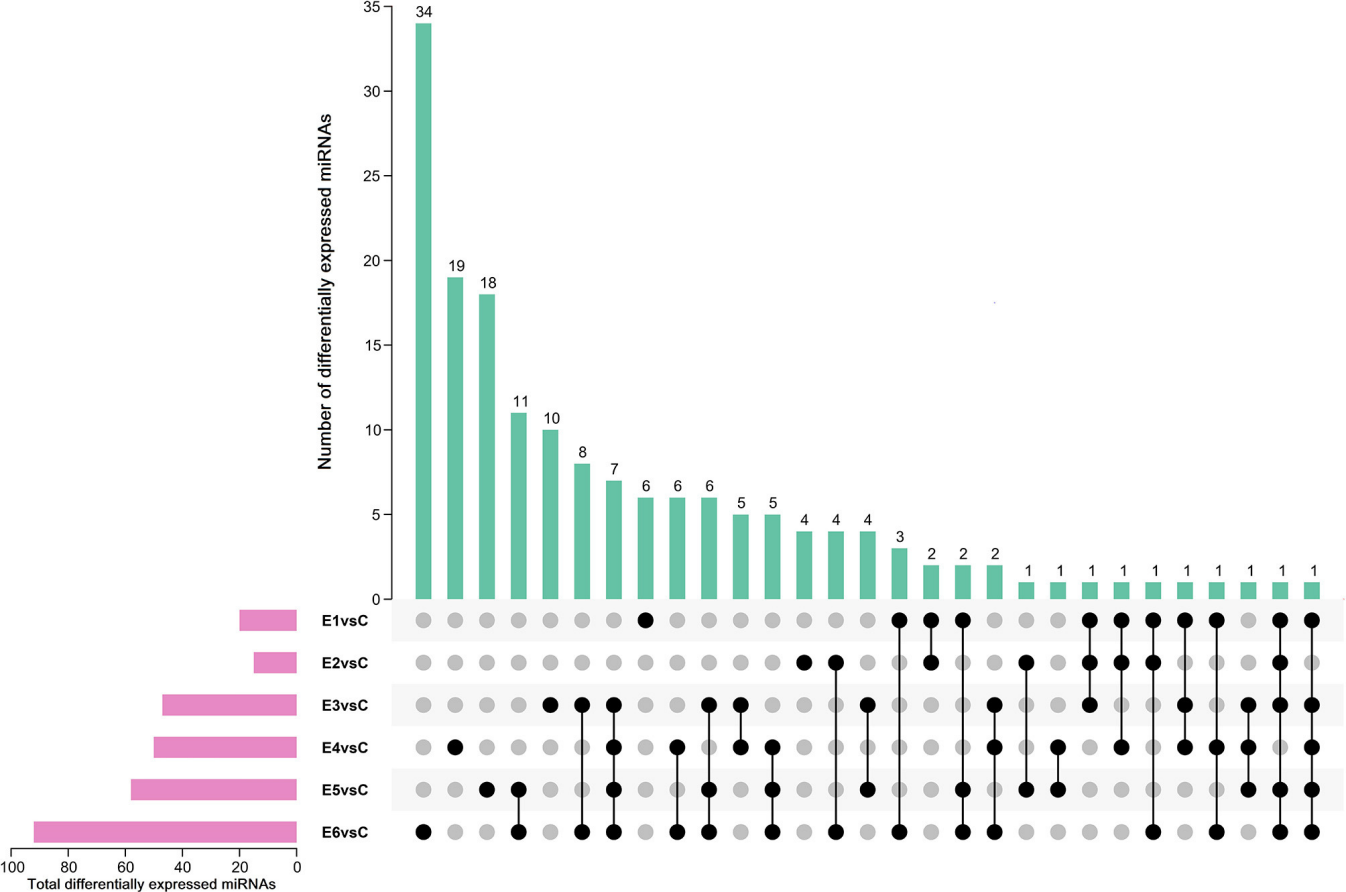
Upset plot diagram of differentially expressed miRNAs (|log_2_ (fold-change)| > 0.5, *p* < 0.05). The number of differentially expressed miRNAs among comparisons of E1 vs Ctrl, E2 vs Ctrl, E3 vs Ctrl, E4 vs Ctrl, E5 vs Ctrl and E6 vs Ctrl.

**Figure 5:**
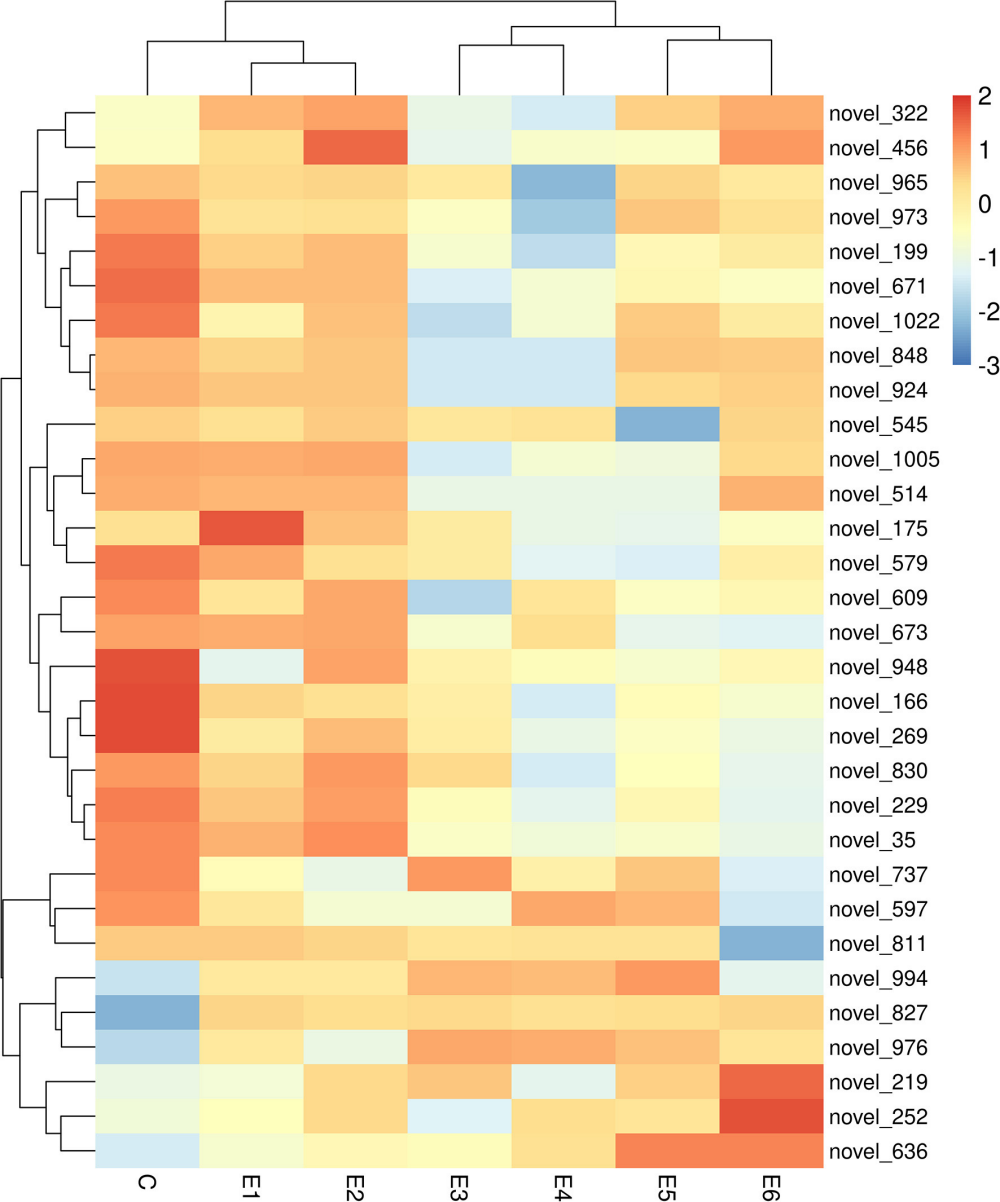
Heatmap of 31 differentially expressed novel miRNA for miRNA sequencing among different group. All these novel miRNA are differentially expressed in more than one group (|log_2_ (fold-change)| >1, *p* < 0.01).

The alignment analysis yielded 150 known miRNAs and 406 novel miRNAs from all groups. Bioinformatic data from high-throughput miRNA sequencing was used to analyze chromosomal localization. The results indicated that 398 novel miRNAs were located on chromosomes, and 8 differentially expressed miRNAs were located in the scaffolds (Supplementary Figure 1). Among these chromosomes, chromosome 3 has 49 novel miRNAs, which was the highest among all chromosomes. On the contrary, chromosome 23 has 4 novel miRNAs, which was the lowest among all chromosomes. Identification of the differentially expressed miRNAs were performed based on the results of the DESeq R package (1.8.3), revealing 282 miRNAs (|log_2_ fold-change |>0.5 and *p* < 0.05) showing significantly altered expressions in the vaccinated groups compared with those of the control group. Moreover, 94 novel miRNAs were screened in the above 282 differentially expressed miRNAs. The number of differentially expressed miRNAs increased gradually with time, and most of them were downregulated. At 90 dpi, 92 differentially expressed miRNAs were identified, compared with the control group (Figure 3). The numbers of unique differentially expressed miRNAs in each group were 6, 4, 10, 19, 18 and 34 (Figure 4). When the thresholds were |log_2_ fold-change |>1 and *p* < 0.01, we identified 48 differentially expressed miRNAs, of which, 31 novel differentially expressed miRNAs are shown in the heatmap (Figure 5). Thirteen novel miRNAs, which were differentially expressed in more than one group, were further analyzed for RT-qPCR verification.

### RT-qPCR Validation of Novel miRNAs

**Figure 6:**
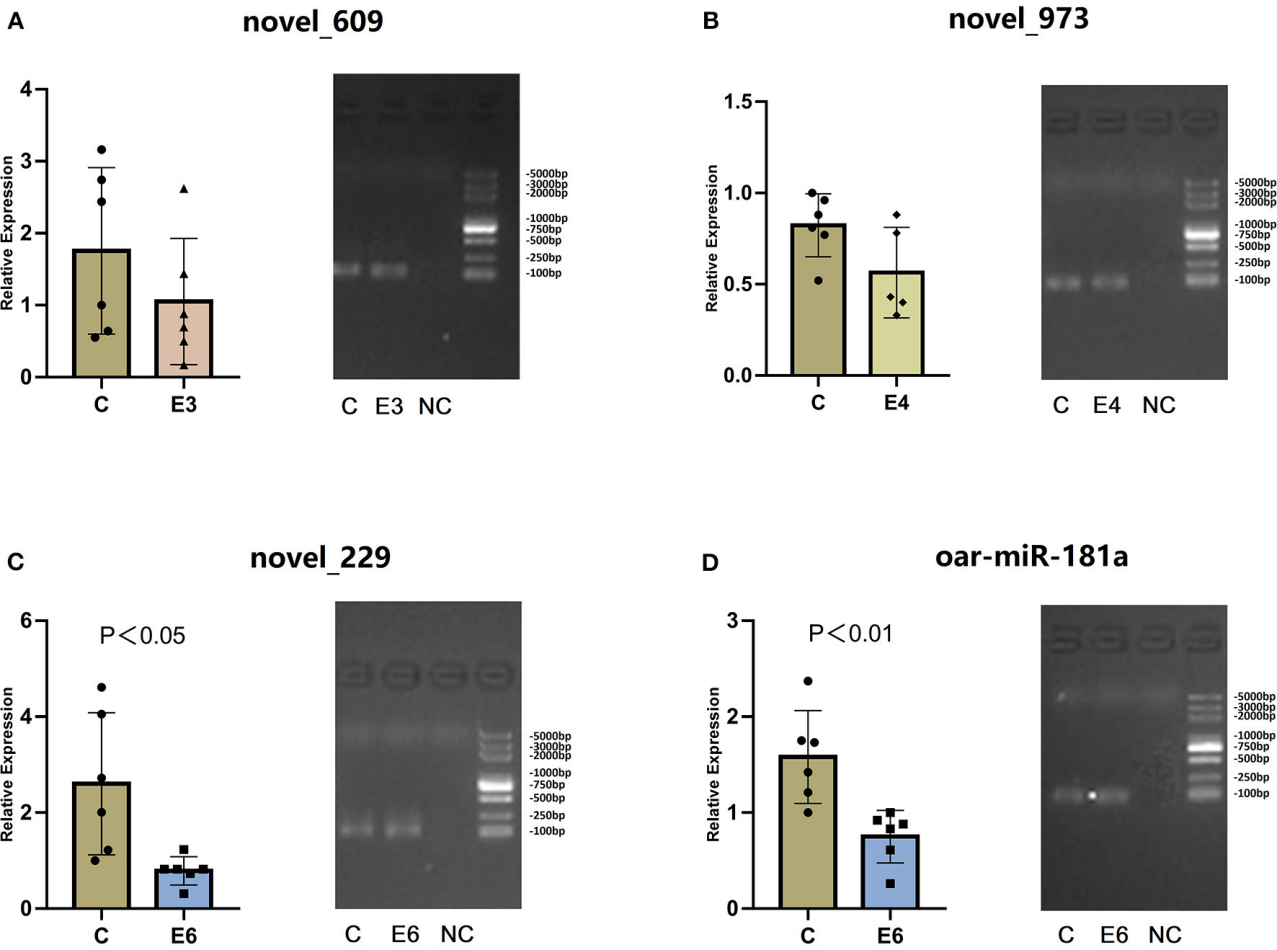
**(A–D)** The relative expression and RT-qPCR products agarose gel electrophoresis of three novel miRNAs and oar-miR-181a. In gel electrophoresis image, from left to right is the control group **(C)**, the experiment groups (E3, E4 and E6) and the negative control group (NC). The marker is D2000 plus DNA ladder, which consists of 8 individual DNA fragments: 100, 250, 500, 750, 1000, 2000, 3000 and 5000 bp.

To verify the RNA-seq results and identify the existence of novel miRNAs, 13 novel miRNAs (|log_2_ fold-change |>1 and *p* < 0.01), which were differentially expressed in more than one group, were selected for RT-qPCR. Table 3 shows the mature miRNA sequences and primer information. Previous research revealed that mmu-miR-181a was differentially expressed during *Brucella abortus* infections (14). In the present study, we selected oar-miR-181a for RT-qPCR validation as a marker miRNA. The RT-qPCR results demonstrated that three novel miRNA (novel_229, novel_609 and novel_973) and oar-miR-181a were consistent with the miRNA-seq results (Figure 6). After vaccination, the expression of novel_229 decreased from 7 dpi to 90 dpi, as well as the novel_973 expression level decreased from 7 dpi to 30 dpi and then increased at 60 dpi. The expression of novel_609 peaked at 14 dpi and maintained a lower level from 21 dpi to 90 dpi (Supplementary Figure 2).

**Table 3:** The RT-qPCR primers sequences of miRNAs.

**No**.	**Name**	**Forward primer sequence (5^′^-3^′^)**	**Reverse primer sequence (5^′^-3^′^)**
1	novel_199	GCGAAACCCTGAACGAAGG	AGTGCAGGGTCCGAGGTATT
2	novel_219	CGACCAGCTGGACTGGGG	AGTGCAGGGTCCGAGGTATT
3	novel_229	CGCTCTTGGGCTCGGATCT	AGTGCAGGGTCCGAGGTATT
4	novel_269	GCGTCTGCCTGGCTCTGTC	AGTGCAGGGTCCGAGGTATT
5	novel_35	TATATTAACTCGGCGTGGCGTCGG	AGTGCAGGGTCCGAGGTATT
6	novel_456	ATATATTTACCCGCGGGGGGCGC	AGTGCAGGGTCCGAGGTATT
7	novel_514	GCGTCCGCCTCTCCTCAG	AGTGCAGGGTCCGAGGTATT
8	novel_579	GCGCGAAAACCTTGATGAACT	AGTGCAGGGTCCGAGGTATT
9	novel_597	GCGCTTAGCCAGGGGCT	AGTGCAGGGTCCGAGGTATT
10	novel_609	ATATTACGCGCTCGGGGCTGCA	AGTGCAGGGTCCGAGGTATT
11	novel_636	GCGCGCGTATGGATGTGTA	AGTGCAGGGTCCGAGGTATT
12	novel_965	GCGCTCATGGCTGGTGG	AGTGCAGGGTCCGAGGTATT
13	novel_973	GCGCGAGAAACTCAAATGAACT	AGTGCAGGGTCCGAGGTATT
14	oar-miR-181a	CGAACATTCAACGCTGTCG	AGTGCAGGGTCCGAGGTATT
15	U6	CTCGCTTCGGCAGCACA	AACGCTTCACGAATTTGCGT

### Functional Analysis of Novel miRNAs

**Figure 7:**
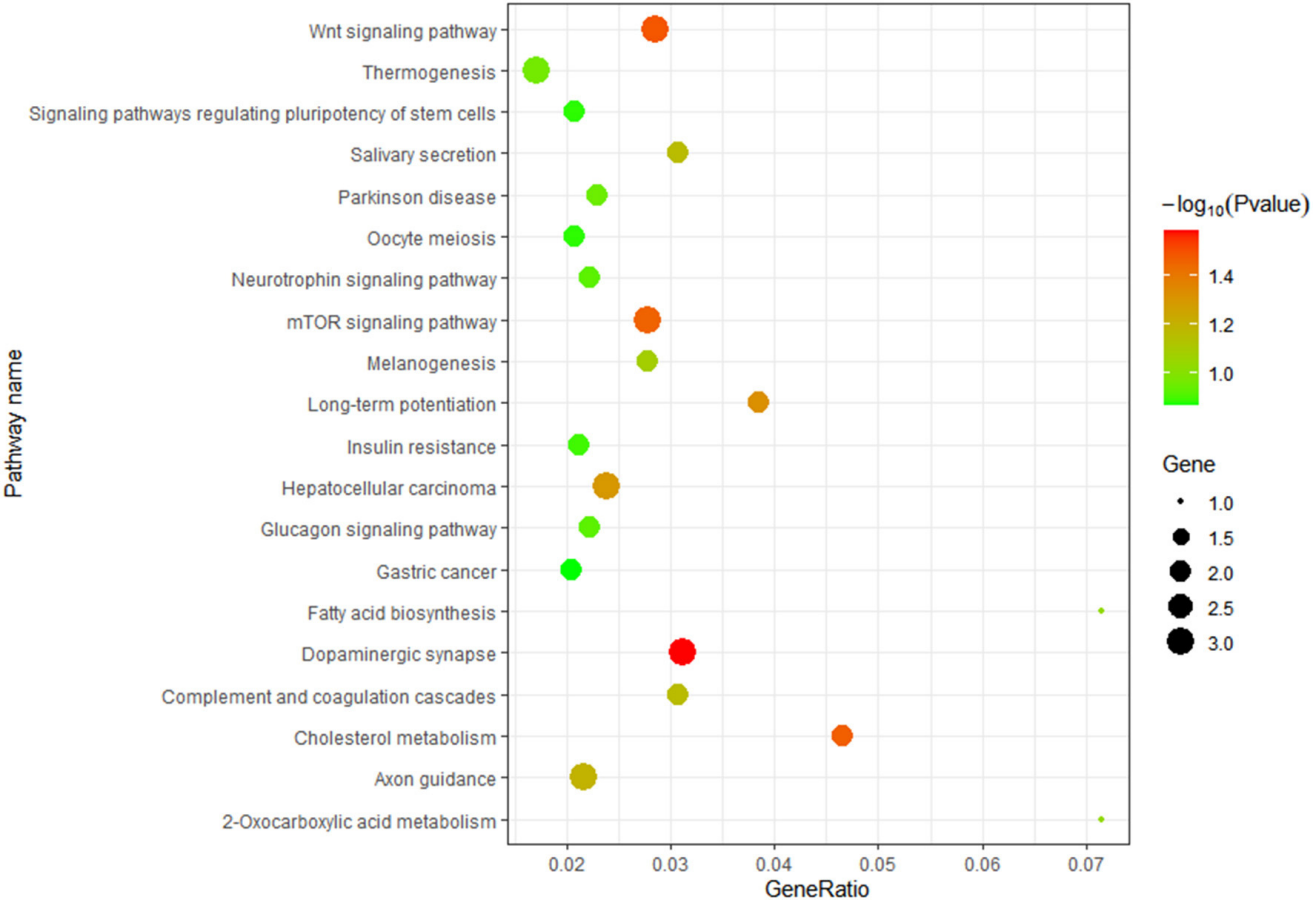
KEGG enrichment analysis. The target genes of novel_229, novel_609, novel_973 and oar-miR-181a were selected.

**Figure 8:**
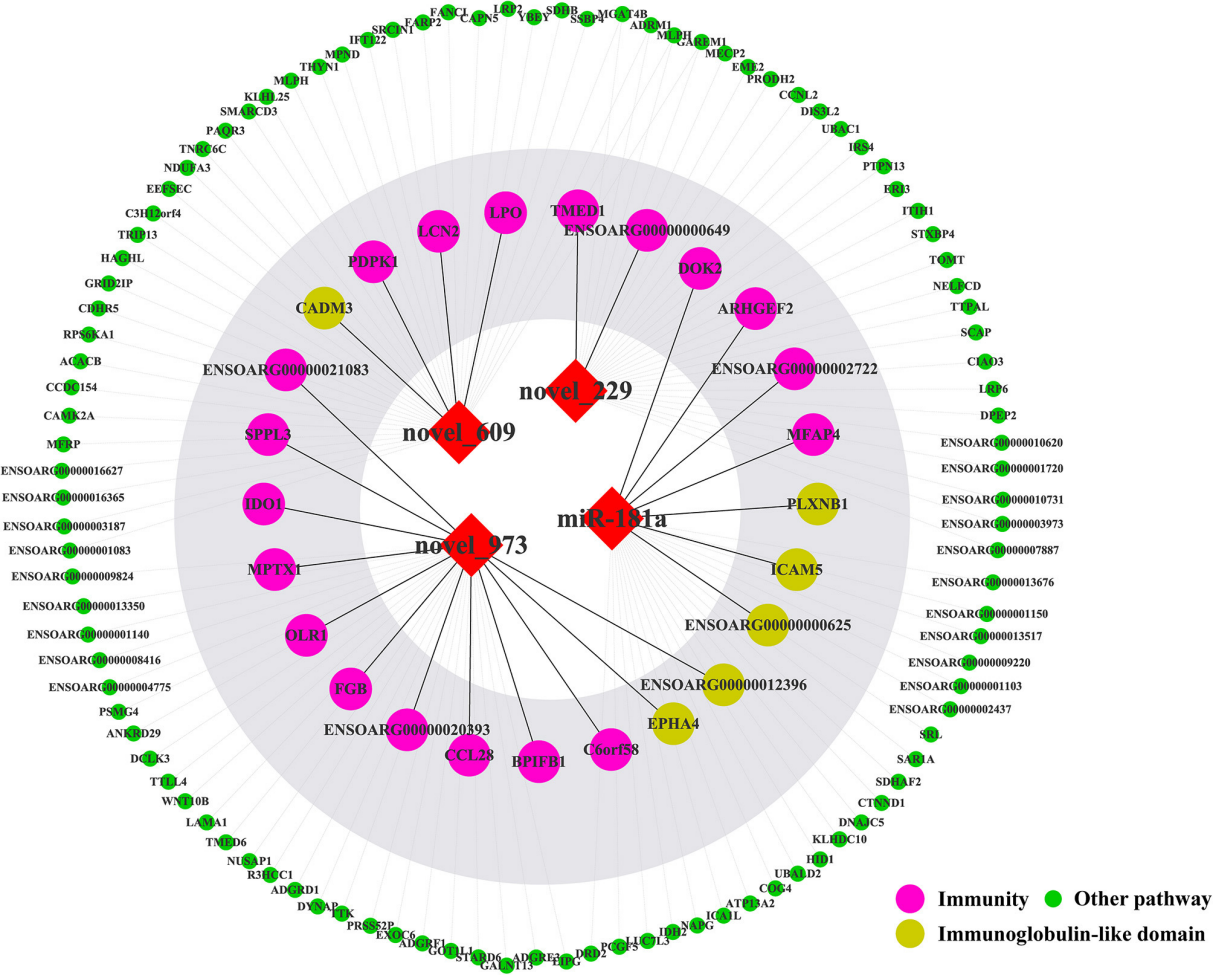
The target genes of four miRNAs. The red diamonds represent miRNAs. The colored circles represent target genes.

TargetScan, miRanda and RNAhybrid were used to predict the target genes of novel_229, novel_609, novel_973 and oar-miR-181a. A total of 131 target genes were obtained and were enriched via the GO and KEGG pathway analyses. GO analysis was performed using DAVID at three levels: biological process, cellular component and molecular function (Supplementary Figure 3). Figure 7 outlines the top 20 significantly enriched KEGG pathways, with the Wnt signaling pathway and the complement and coagulation cascades being the immune pathways. The mTOR signaling pathway negatively regulates autophagy, which is essential for the intracellular survival of *Brucella*. As shown in Figures 8, 25 target genes were involved in the immune responses. The immune-related target genes of novel_229 included *TMED1* and *ENSOARG00000000649*. *LCN2, PDPK1* and *LPO* were the immune-related target genes of novel_609. The immune-related target genes of novel_973 were *C6orf58, SPPL3, BPIFB1, ENSOARG00000021083, MPTX1, CCL28, FGB, IDO1, OLR1* and *ENSOARG00000020393*. Four target genes of oar-miR-181a were associated with the immune response, including *ENSOARG00000002722, ARHGEF2, MFAP4 and DOK2*. Moreover, 6 target genes encoded the immunoglobulin-like domains (Figure 8).

### Biological Pathways of the Immune-Related Target Genes

**Figure 9:**
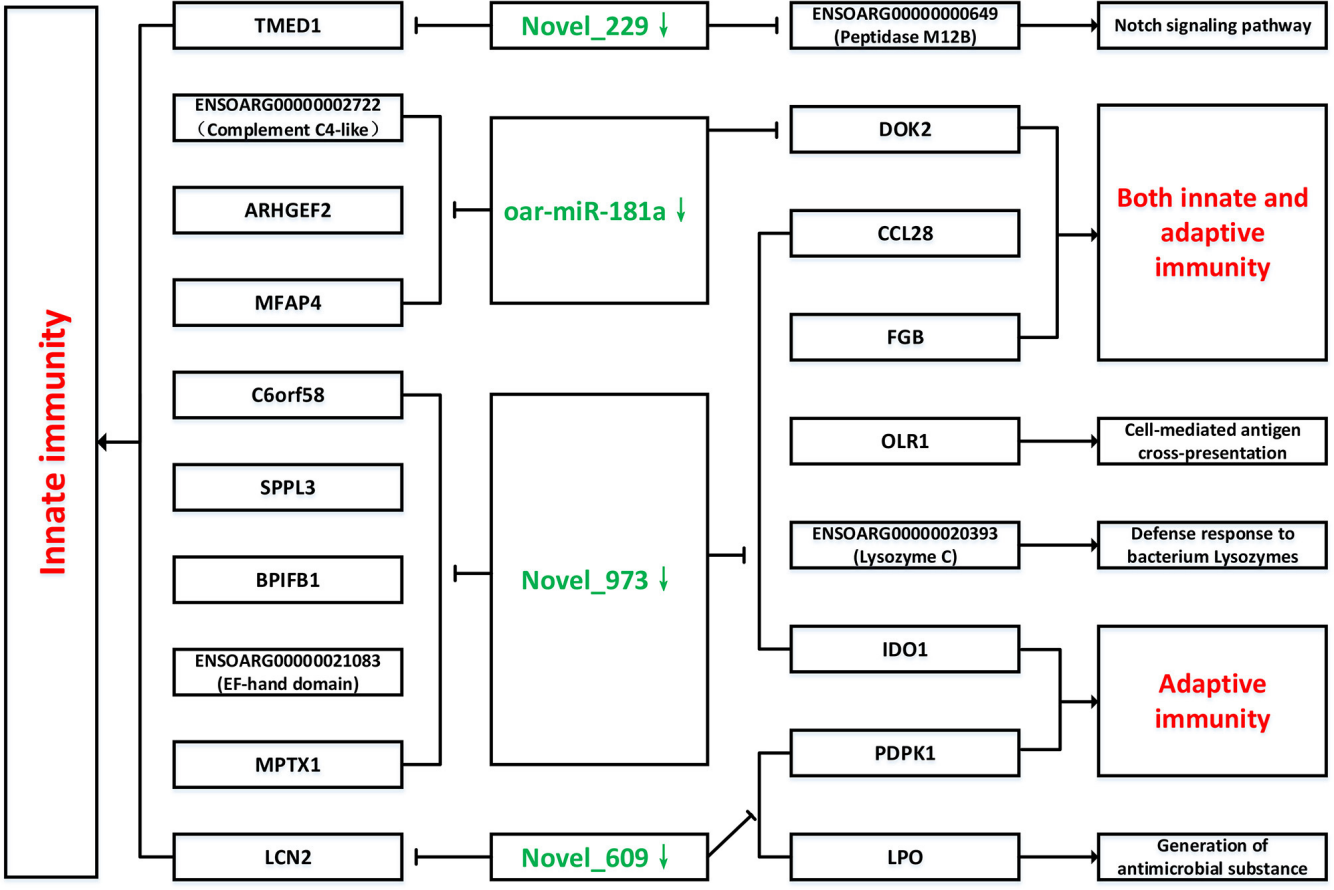
Network of novel_229, novel_609, novel_973, oar-miR-181a and their target genes involved in immunology and inflammation pathways. The green color represent down-regulated miRNAs, as well as red color represent up-regulated immunology pathways.

The above-mentioned immune-related genes were selected, together with the novel_229, novel_609, novel_973 and oar-miR-181a, for further miRNA-mRNA network construction and function analysis. The target genes and their related biological pathways were summarized in Figure 9. These target genes are involved in the innate immunity, adaptive immunity, defense responses to bacteria, and the Notch signaling pathways. In the submandibular lymphatic nodes of the sheep inoculated with the *B.suis* S2 vaccine, ten miRNA-mRNA network-associated innate immunity pathways were predicted. *DOK2, CCL28* and *FGB* were associated with both the innate and adaptive immunity pathways. PDPK1 and IDO1, which were regulated by novel_609 and novel_973, were involved in the adaptive immunity pathways. The target genes of novel_229 and novel_609 were involved in the Notch signaling pathway and antimicrobial generation, respectively.

## Discussion

Brucellosis is a globally distributed zoonotic bacterial disease caused by the *Brucellaceae* family in the alpha-2 subclass of *Proteobacteria. B. melitensis* is an etiological agent that mainly affects goats and sheep and leads to substantial economic losses in the animal industry, such as decreased fertility, loss of young, and decreased milk production. *B. melitensis* is also the most virulent species in humans and it significantly affects human health. *B.suis* S2 is extensively used as an oral vaccine in China to prevent ovine and caprine brucellosis. Mice are the premier mammalian model system for *Brucella*-induced immunity research because of their close genetic and physiological similarities to sheep and the ease by which the mouse genome can be manipulated and analyzed (6). However, the use of mouse models may influence the identification of sheep-specific immune response genes and clinical markers for the diagnosis and prognosis of brucellosis. The study on the immunological mechanisms of vaccine-induced protection against *Brucella* in sheep remains comparatively rare. Identifying functional molecules involved in the host immune response will deepen our understanding of further research on optimizing vaccines and developing molecular diagnostic targets.

MiRNAs have been proven to participate in brucellosis immunopathogenesis. Our previous studies found that the autophagic pathway associated miRNAs, mmu-miR-146b-5p and mmu-miR-149-3p was up-regulated and down-regulated in RAW264.7 macrophage cells infected with *B.melitensis* M5-90 mutant strain, respectively (18, 19). In *B.melitensis*-infected goat fibroblasts, we found that miR-744, miR-29a-5p and miR-193b-5p play pleiotropic roles in inflammatory and immune responses (20). To uncover the host miRNA-driven defense mechanism in sheep, we investigated the *in vivo* changes in sheep immunized with *B.suis* S2. The sheep were orally immunized with 1 mL of diluted *B.suis* S2 (2 × 10^10^ CFU) in a biosafety level III facility. All animals were monitored daily for 90 consecutive dpi. Fiebig et.al demonstrated that *B. abortus* introduced conjunctivally could be detected in the retropharyngeal lymph nodes of cattle between 7 and 21 dpi (31). In our study, we collected the submandibular lymphatic nodes of sheep seropositive for *Brucella* at 7, 14, 21, 30, 60, and 90 dpi. MicroRNA sequencing revealed that 282 differentially expressed miRNAs were significantly enriched in immune pathways, especially in innate and adaptive immunity pathways. According to the alignment results and RT-qPCR experiment, three novel differentially expressed miRNAs were identified: novel_229, novel_609 and novel_973. After immunization with *B.suis* S2 vaccine, the expressions of these miRNAs declined at least twice. The expressions of novel_229 and novel_973 decreased from 7 dpi to 30 dpi. At 60 dpi, the expression level of novel_229 continued to decrease, as well as novel_973 increased. The expression of novel_609 peaked at 14 dpi and maintained a lower level from 21 dpi to 90 dpi. The IgG antibodies peaked by day 14 after the first immunization and remained the level from 14 dpi to 90 dpi. The correlation between novel miRNAs expression and antibody levels needs to be validated prospectively. Functional analyses of these miRNAs revealed that they participated in immune response pathways, including the innate and adaptive immunity pathways. Notably, nine target genes of these novel miRNAs were involved in the innate immunity.

The immune-related target genes of novel_229 included *ENSOARG00000000649* and *TMED1*. *ENSOARG00000000649* encoded peptidase M12B domain, which was associated with the Notch signaling pathway. Several studies have shown that during bacterial infections, Notch signaling pathways modulate the CD4^+^ T cells' function and regulate T cell polarization (32). The protein encoded by *TMED1* is a member of the p24 family of trafficking proteins, and recent research revealed that TMED1, RNF26, TMEM43, TMEM33 and ENDOD1 form a complex that modulates innate immune signaling through the cGAS-STING pathway (33).

*LPO, LCN2* and *PDPK1* were immune-related target genes of novel_609. *LPO* gene encodes a member of the peroxidase family of proteins which catalyzes the generation of the antimicrobial substance hypothiocyanous acid (34). Lipocalin-2 (Lcn2), also known as neutrophil gelatinase-associated lipocalin, is an innate immune protein that skews different types of macrophages toward M1 activation and induces the expressions of proinflammatory cytokines (e.g., IL-1β, IL-6, and TNF-α) in response to lipopolysaccharide stimulation by activating the NF-κB-STAT3 loop (35). *PDPK1* encodes 3-phosphoinositide-dependent protein kinase-1, which is associated with PI3Kδ inhibition, consistently improved function and significantly increased bacterial killing of neutrophils (36).

The immune-related target genes of novel_973 included *CCL28, FGB, C6orf58, SPPL3, BPIFB1, ENSOARG00000021083, MPTX1, OLR1, ENSOARG00000020393* and *IDO1*. Two genes (*CCL28* and *FGB*) were associated with both innate and adaptive immunity pathways. *CCL28* encodes CCL28, a cellular component chemokine (β-chemokine) and drives the mucosal homing of T and B lymphocytes (37). This chemokine plays dual roles in antimicrobial and immunomodulatory properties. High CCL28 expression levels provide a constitutive innate immune defense against various bacterial pathogens and strengthen the protein's involvement in antimicrobial activity (38). *FGB* encodes the fibrinogen beta chain, which is associated with host defense at an early stage of the infectious process and facilitates antibacterial immune responses via both innate and T-cell-mediated pathways (39, 40). Fibrinogen plays a key role in determining the outcomes of sepsis, and higher fibrinogen levels are associated with better outcomes (41). In our study, *C6orf58, SPPL3, BPIFB1, ENSOARG00000021083* and *MPTX1* may have been induced by the downregulation of novel_973, suggesting that host innate immunity was activated after immunization with *B. suis* strain S2. Signal peptide peptidase-like 3 (SPPL3) is a member of the intramembrane aspartyl protease family, which functions in the endoplasmic reticulum in T cells to promote store-operated calcium entry in response to T-cell receptor engagement by enhancing the interaction between STIM1 and Orai1 (42, 43). Bactericidal/permeability-increasing (BPI)-fold-containing family B member 1 (BPIFB1) belongs to the BPI-fold-containing family and is considered to contribute to innate immunity through its structural similarity with BPI protein and LPS-binding protein, both of which are innate immune molecules with recognized roles in sensing and responding to Gram-negative bacteria (44). The target gene *IDO1* of novel_973 encodes an intracellular enzyme that catalyzes the early stage of tryptophan catabolism along the kynurenine pathway. IDO-1 affects immunity through two nonexclusive mechanisms: establishment of a local response with amino acid deprivation, which inhibits pathogen growth, and production of tryptophan metabolites with immunomodulatory functions or cytotoxic agents that inhibit T-cell activation and modulate differentiation of naive T cells into regulatory T cells (45).

Moreover, previous research found that miR-181 expression *in vivo* led to substantial increases in both B-lymphoid (CD19^+^) cell and cytotoxic T-cell (CD8^+^) development in the thymus (46). Members of the miR-181 family have been shown to be dynamically regulated depend on the T-cell activation stage (15). During *B. suis* infection, miR-181a-5p expression was induced in porcine and murine macrophages, suggesting that miR-181a-5p may be involved in adaptive immunity (16). However, in our experiment, oar-miR-181a expression was decreased after immunizing sheep with *B. suis* strain S2, which requires further confirmation with additional technologies. Four target genes of oar-miR-181a participated in innate immunity, which were *ENSOARG00000002722, ARHGEF2, MFAP4* and *DOK2. ENSOARG00000002722* encodes complement C4-like protein, which is the key molecule in the complement system. Complement C4 is one of the chief constituents of innate immunity for immediate recognition and elimination of invading microbes, and plays an essential role in the functions of both classical and lectin complement pathways (47). *ARHGEF2* encodes the microtubule-associated immune molecule guanine nucleotide exchange factor-H1 (GEF-H1), which is crucial in coupling microtubule dynamics to the initiation of microtubule-mediated immune responses (48). Microfibril-associated glycoprotein 4 (MFAP4), a pattern recognition-like molecule with a fibrinogen-like domain (FBG), has the ability to recognize and agglutinate pathogens, playing an essential role in host innate immune defense (49, 50). Dok2, which belongs to the protein tyrosine kinases family, is the negative regulator of innate immunity in the early inflammatory responses to lipopolysaccharide *in vivo* (51). These results enable better understanding of the immune responses of sheep upon *B.suis* S2 vaccination and the roles of miRNAs in host innate and adaptive immunity, which provides valuable information for preventing brucellosis.

## Data Availability Statement

The datasets presented in this study can be found in online repositories. The names of the repository/repositories and accession number(s) can be found below: https://www.ncbi.nlm.nih.gov/, PRJNA775839.

## Ethics Statement

The animal study was reviewed and approved by The Academic Committee of the College of Animal Science and Technology of Hainan University.

## Author Contributions

FW, LD, and WZ conceived the experiments. SC and CW conducted the experiments and analyzed the results. DZ, GL, and XW provided the laboratory support and vaccine immunization. YL, SZ, SF, XH, BY, and QZ contributed reagents. QC, CM, and ZZ contributed strains and materials. QA and YC uploaded RNA-seq data. The manuscript was written and reanalyzed by SC. All authors have approved the final manuscript.

## Funding

This work was financially supported by Hainan Provincial Natural Science Foundation (No.2019RC123), Natural Science Foundation of China (32160831), China Agriculture Research System of MOF and MARA (CARS-38), Academician Innovation Platform Project of Hainan Province (No. YSPTZX202013), and Inner Mongolia Major Scientific and Technological Special Project (2019ZD016, 2019GG363, and 2020ZD0003).

## Conflict of Interest

DZ, GL, and XW were employed by Jinyu Baoling Bio-pharmaceutical Co., Ltd. The remaining authors declare that the research was conducted in the absence of any commercial or financial relationships that could be construed as a potential conflict of interest.

## Publisher's Note

All claims expressed in this article are solely those of the authors and do not necessarily represent those of their affiliated organizations, or those of the publisher, the editors and the reviewers. Any product that may be evaluated in this article, or claim that may be made by its manufacturer, is not guaranteed or endorsed by the publisher.
