# Supplementary material for: Downregulation of Three Novel miRNAs in the Lymph Nodes of Sheep Immunized With the Brucella suis Strain 2 Vaccine
Source: Front Vet Sci. 2022 Feb 22;9:813170. doi: 10.3389/fvets.2022.813170 (PMC8902169; doi:10.3389/fvets.2022.813170)
Supplement: Supplementary file 1 [file Data_Sheet_1.docx]

**Table S1** **The effective reads in each sample.**

| **sample** | **raw_reads** | **clean_reads** | **clean_bases** | **error_rate(%)** | **Q20(%)** | **Q30(%)** | **GC_content(%)** |
| --- | --- | --- | --- | --- | --- | --- | --- |
| C_379_HY | 87626536 | 84271060 | 12.64G | 0.02 | 98.29 | 94.99 | 47.96 |
| C_379_HZ | 99433208 | 98623686 | 14.79G | 0.03 | 97.88 | 93.96 | 54.78 |
| C_511_HY | 94497206 | 90380726 | 13.56G | 0.03 | 97.78 | 93.72 | 49.38 |
| C_511_HZ | 1.04E+08 | 1.02E+08 | 15.32G | 0.02 | 98.16 | 94.43 | 45.41 |
| C_587_HY | 94651996 | 92978618 | 13.95G | 0.01 | 97.63 | 93.92 | 50.71 |
| C_587_HZ | 88617104 | 86981960 | 13.05G | 0.01 | 97.53 | 93.7 | 49.48 |
| E1_239_HY | 89293970 | 86615714 | 12.99G | 0.03 | 97.84 | 93.8 | 45.02 |
| E1_239_HZ | 94441936 | 92737346 | 13.91G | 0.01 | 97.56 | 93.94 | 48.71 |
| E1_273_HY | 85809490 | 84175840 | 12.63G | 0.03 | 97.93 | 93.87 | 44.87 |
| E1_273_HZ | 85854068 | 84284072 | 12.64G | 0.02 | 98.1 | 94.25 | 44.69 |
| E1_283_HY | 94088022 | 92861722 | 13.93G | 0.01 | 97.41 | 93.51 | 49.81 |
| E1_283_HZ | 88436830 | 87016994 | 13.05G | 0.01 | 97.35 | 93.5 | 55.89 |
| E2_231_HY | 98518532 | 94304650 | 14.15G | 0.02 | 98.28 | 94.98 | 47.54 |
| E2_231_HZ | 88063428 | 85005962 | 12.75G | 0.01 | 97.88 | 94.43 | 46.74 |
| E2_237_HY | 95218858 | 93774754 | 14.07G | 0.01 | 97.78 | 94.41 | 48.2 |
| E2_237_HZ | 90869724 | 88727096 | 13.31G | 0.03 | 97.75 | 93.56 | 45.52 |
| E2_265_HY | 88657200 | 87808038 | 13.17G | 0.01 | 97.53 | 93.75 | 46.26 |
| E2_265_HZ | 1.15E+08 | 1.12E+08 | 16.76G | 0.03 | 97.82 | 93.89 | 50.99 |
| E3_221_HY | 85872760 | 83532464 | 12.53G | 0.03 | 97.97 | 94.02 | 46.04 |
| E3_221_HZ | 1.01E+08 | 94032930 | 14.1G | 0.02 | 98.04 | 94.78 | 51.32 |
| E3_285_HY | 88661604 | 85960762 | 12.89G | 0.03 | 97.75 | 93.66 | 45.61 |
| E3_285_HZ | 91437816 | 89610884 | 13.44G | 0.03 | 97.93 | 93.94 | 44.94 |
| E3_292_HY | 93097924 | 87325374 | 13.1G | 0.03 | 97.91 | 93.95 | 48.17 |
| E3_292_HZ | 82553078 | 80511352 | 12.08G | 0.03 | 97.69 | 93.49 | 45.79 |
| E4_204_HY | 1.12E+08 | 1.07E+08 | 16.05G | 0.02 | 98.01 | 94.38 | 48.73 |
| E4_204_HZ | 91428466 | 89523970 | 13.43G | 0.02 | 98.3 | 94.76 | 45.03 |
| E4_215_HY | 87175326 | 84329932 | 12.65G | 0.03 | 97.91 | 93.93 | 45.5 |
| E4_215_HZ | 95046396 | 92282300 | 13.84G | 0.03 | 97.82 | 93.81 | 45.62 |
| E4_244_HZ | 98327848 | 96005900 | 14.4G | 0.03 | 97.78 | 93.68 | 45.25 |


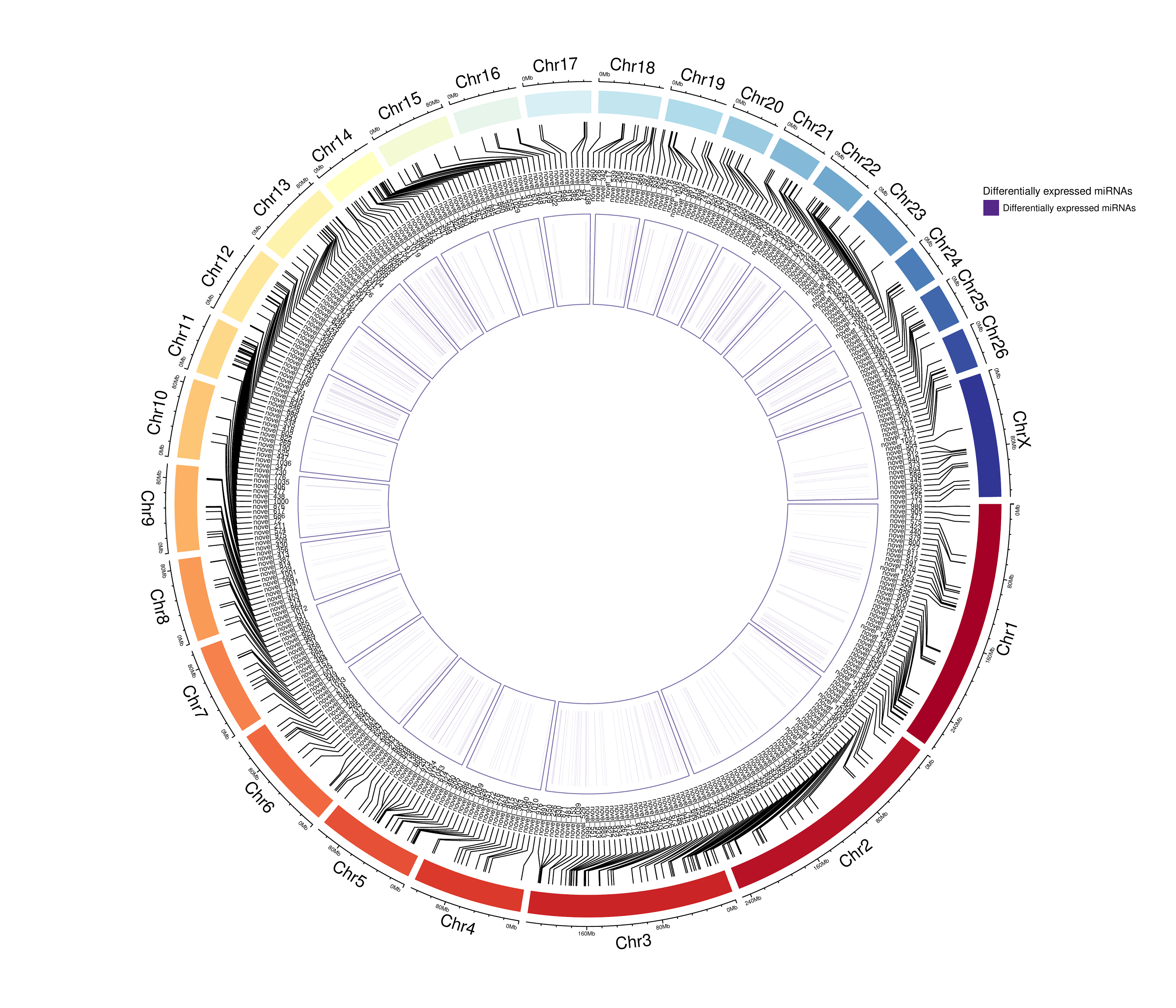


**Figure S1.** Novel miRNAs distribution among *Ovis aries* genome. The blue line represents the location of novel miRNAs.


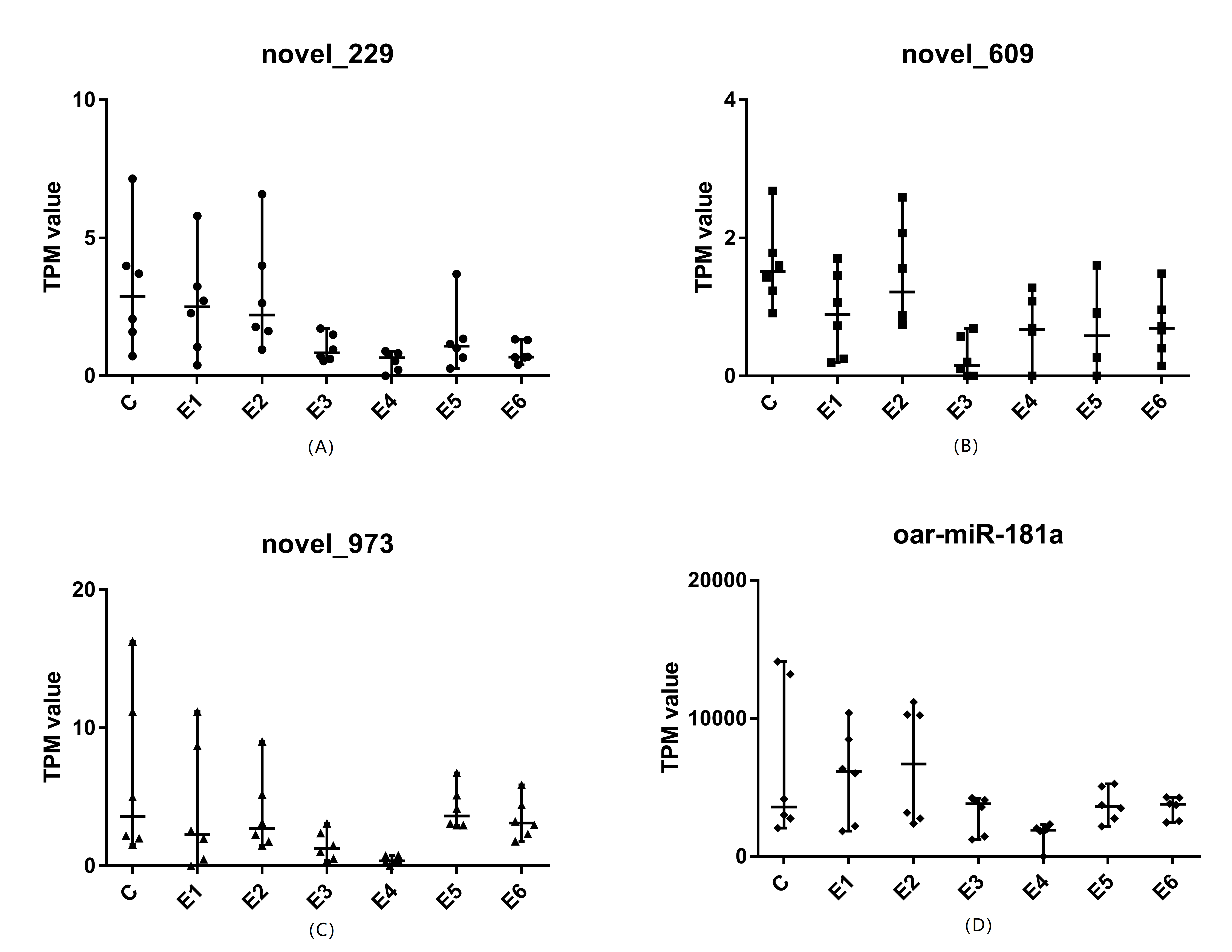


**Figure S2.** The relative expression level (TPM value) of three novel miRNAs and oar-miR-181a at all time points.

**
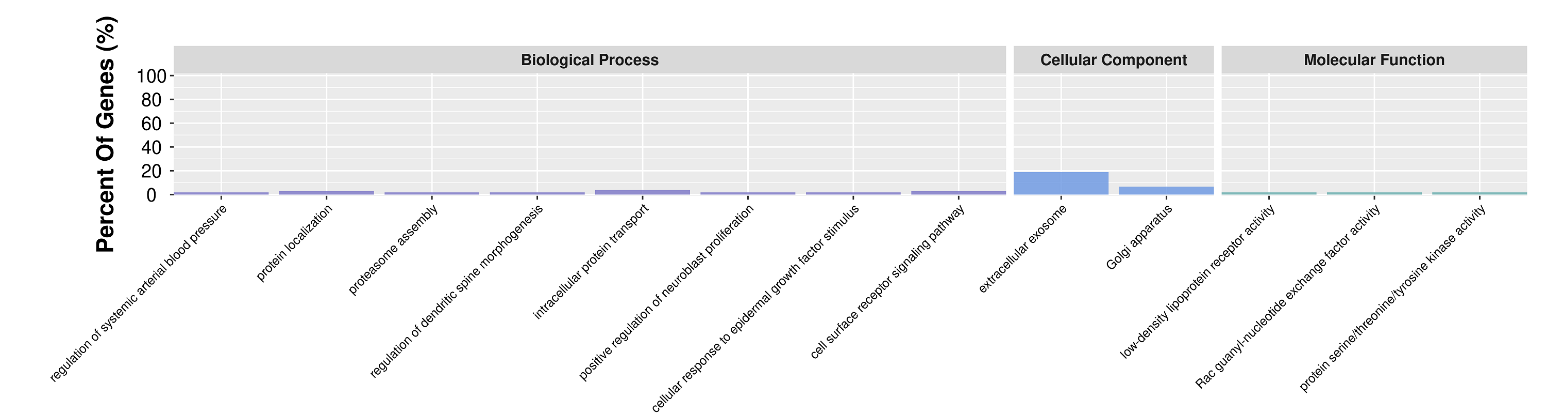
**

**Figure S3.** GO enrichment analysis. The target genes of novel_229、novel_609、novel_973 and oar-miR-181a were selected.
