# Supplementary figures and images for: Downregulation of Three Novel miRNAs in the Lymph Nodes of Sheep Immunized With the Brucella suis Strain 2 Vaccine
Source: Front Vet Sci. 2022 Feb 22;9:813170. doi: 10.3389/fvets.2022.813170 (PMC8902169; doi:10.3389/fvets.2022.813170)

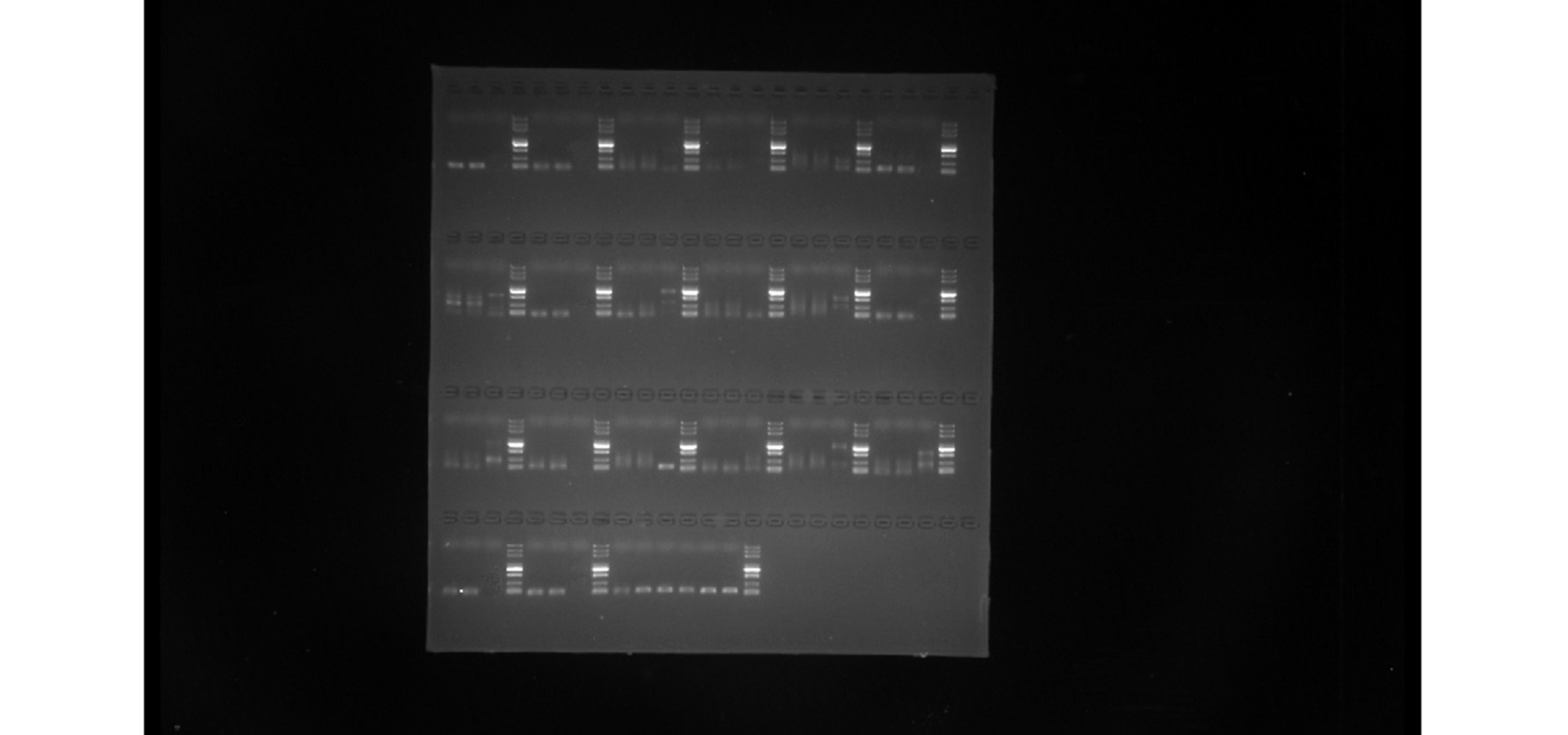

Supplement: Supplementary file 3 [file Image_1.JPEG]
